# Advanced Digital Imaging Assessment Method for Testing Surface Fuzzing in Textile Materials

**DOI:** 10.3390/polym18121532

**Published:** 2026-06-19

**Authors:** Juro Živičnjak, Antoneta Tomljenović, Maja Somogyi Škoc, Željko Penava

**Affiliations:** 1 Department of Materials, Fibers and Textile Testing, University of Zagreb Faculty of Textile Technology, Prilaz baruna Filipovića 28a, 10000 Zagreb, Croatia; juro.zivicnjak@ttf.unizg.hr (J.Ž.); maja.somogyi@ttf.unizg.hr (M.S.Š.); 2 Department of Textile Design and Management, University of Zagreb Faculty of Textile Technology, Prilaz baruna Filipovića 28a, 10000 Zagreb, Croatia; zeljko.penava@ttf.unizg.hr

**Keywords:** textile testing, fuzzing assessment, digital imaging, advanced method, innovative apparatus, woven fabric, polymer–based fibers

## Abstract

Textile materials made from staple fibers typically have protruding fibers on their surface, commonly referred to as surface hairiness. During fraying, the surface of the textile material is susceptible to damage, which affects its appearance and leads to fuzzing by roughening or the emergence of new fibers. The propensity for fuzzing is assessed using the standard visual method (EN ISO 12945-4:2020), which is intuitive and cost-effective but better suited for evaluating more pronounced surface phenomena, such as pilling. This is mainly because fuzzing is usually accompanied by pilling, and their simultaneous occurrence makes separate analysis difficult. As a result, instrumental methods for assessing fuzzing that provide a more objective evaluation are rarely reported. In this research, an advanced digital imaging assessment method was introduced, using an innovative apparatus that, with simultaneous assessment of pilling, enabled separate digital imaging of the same textile fabric specimen’s surface fuzzing through a refined viewing angle. Additionally, newly developed software enabled digital analysis and acquisition of quantitative numerical values related to surface fuzzing. The research was conducted on six single-component woven fabrics made from cotton, wool, viscose, polyamide 6.6, polyester, and acrylic. Fuzzing was induced using an ICI tester (EN ISO 12945-1:2020) and a Martindale tester (EN ISO 12945-2:2020) through predefined box revolutions and fuzzing rubs ranging from 125 to 30,000. Fuzzing was assessed using both the standard visual method and the advanced digital imaging assessment method, with grading according to established classes based on the percentage change in fuzzing layer height. The results highlight the applicability of the advanced digital assessment method, as it separately captures the occurrence of fuzzing and distinguishes it from pilling.

## 1. Introduction

**Figure 1:**
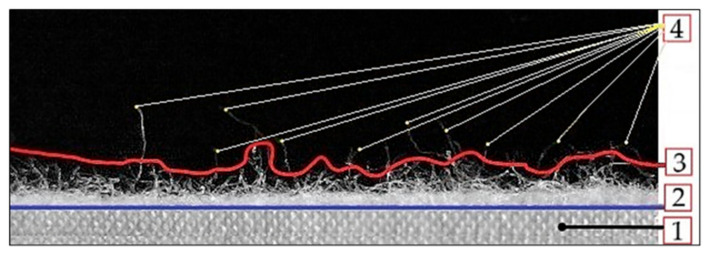
Side view of woven fabric surface: 1—woven fabric layer; 2—interface line between the two layers, marking the end of the woven fabric layer and the beginning of the protruding fibers layer; 3—end of the protruding fibers layer; 4—protruding fibers of unusual height.

Textile materials are inhomogeneous structures composed of interconnected fibers with a characteristic three-dimensional surface. Many fiber ends protrude from the surface, which is especially common in textiles made from staple fibers. These protruding fiber ends of varying heights form a layer on the fabric surface (Figure 1), known as surface hairiness [1].

The surface hairiness of woven fabrics primarily originates from the properties of the polymer-based fibers used in their production [2]. Fabrics made from natural cotton and wool fibers typically exhibit relatively high surface hairiness due to the inherent characteristics of these fibers: cotton’s moderate fiber length, crimp, and variability in fineness, and wool’s natural crimp, scale structure, and variability in fiber length, all of which promote fiber protrusion [3,4]. Fabrics made from viscose, a regenerated cellulose fiber with a higher tendency to fibrillation, generally produce moderate to high levels of hairiness, especially in staple form [5]. Fabrics made from synthetic staple polyamide 6.6 and polyester fibers tend to exhibit lower surface hairiness due to the high tensile strength, low crimp, smooth surface morphology, and low bending rigidity of these fibers [6,7]. In contrast, fabrics made from synthetic acrylic staple fibers of moderate fineness typically show higher surface hairiness because of their wool-like crimp and tendency to shed fibers [8].

In addition to fiber properties, the surface hairiness of fabrics is influenced by the yarn construction used in their production, fabric structural properties, and surface finishing treatments. Yarn construction and its mechanical properties are largely determined by the spinning method, which controls fiber orientation within the yarn. The most commonly used are ring-spun yarns [9], which exhibit high yarn hairiness due to relatively poor alignment and loose wrapping of the fibers [10]. Fabric structure, including weave type, the number of warp and weft yarns per unit area, and crimp, significantly affects surface hairiness. Tighter weaves or a higher number of warp and weft yarns per centimeter generally reduce hairiness by better binding protruding fibers and creating a more compact surface, while looser or more open structures allow greater fiber protrusion and increased hairiness [11]. Surface finishing treatments can also significantly alter fabric surface hairiness, as mechanical or thermal processes such as singeing reduce fabric hairiness and produce a smoother, cleaner surface [12].

During use and care (e.g., washing, drying, ironing) fabric surfaces are prone to mechanical damage, most commonly caused by abrasion. Abrasion is a progressive wear process resulting from repeated frictional contact and rubbing, which leads to the gradual erosion of the fabric surface, fiber damage, and eventual material loss. It affects both the surface appearance and internal structure of the fabric by roughening protruding fibers, causing fiber breakage, fibrillation, and the pull-out of individual fibers or fiber bundles from the yarn structure [13]. Abrasion initially causes roughening of existing protruding fibers on the fabric surface and the appearance of new protruding fibers, a phenomenon known as fuzzing [14]. With prolonged abrasion, in addition to fuzzing, the protruding fibers become entangled into dense clusters (pills) that block light and cast shadows, a phenomenon known as pilling. Although fuzzing generally precedes pilling, the two phenomena usually coexist on the fabric surface [14]. Both fuzzing and pilling are considered undesirable because they deteriorate the mechanical properties, aesthetic appeal, and overall usability of fabrics [15].

The extent and intensity of fabric surface damage depend on the underlying formation mechanisms, which result from contact forces and abrasion mechanics. In practice, these forces typically include a normal load acting perpendicular to the surface and a frictional force acting tangentially, opposing relative motion. This interaction generates transverse compressive and axial shear stresses within the fiber near the fabric surface. High subsurface shear stress can occur and cause internal cracks in fibers of various forms due to a reduced fiber cross-section, as well as inter-fiber abrasion within the yarns and fabric structure, where splitting may develop into a highly fibrillated end or a fiber-angled break [16].

The fabric’s tendency to surface fuzzing and pilling is typically assessed by the standard visual method, as specified in EN ISO (EN ISO 12945-4:2020 [17]) and ASTM standards. The methodology is the same in both and requires that specimens are first abraded using one of the standard abrasion methods defined in separate standards. Both include the Martindale method (EN ISO 12945-2:2020 [18] and ASTM D4970/D4970M-22 [19]) and the random tumble pilling method (EN ISO 12945-3:2020 [20] and ASTM D3512/D3512M-22 [21]). Comparing the methodology of these two Martindale standards, they are essentially the same. The difference lies in the dimensions of the circular samples (140 mm in diameter according to the EN ISO standard and 38 mm in diameter according to the ASTM standard) and the applied load. In addition, the rotating box method (EN ISO 12945-1:2020 [22]) is specific to the EN ISO standards, whereas the brush pilling tester (ASTM D3511/D3511M-16 [23]) and the elastomeric pad method (ASTM D3514/D3514M-16 [24]) are specific to ASTM standards. Unlike conventional abrasion resistance test methods (e.g., EN ISO 12947-2:2016 [25] and ASTM D4966-22 [26]), which apply pressures of 9 or 12 kPa to induce progressive wear and cause material breakdown using the Martindale tester, these abrasion methods are much gentler. They use different abrasion mechanics and types of abradant material, which directly affect the level of surface wear, fiber fibrillation, protrusions, and thus the formation and intensity of fuzzing and pilling [27,28].

The rotating box method [22] relies on gentle random tumbling with primarily fabric face-to-face contact inside a cork-lined chamber, producing relatively mild abrasion. In contrast, the Martindale method [18,19] applies controlled multidirectional rubbing (Lissajous motion) under defined pressure against a standard wool abradant or the fabric itself, resulting in more intense and directional surface damage. The random tumble method [20,21] uses higher-energy tumbling, during which the face and back of the fabric rub against each other and a mildly abrasive liner, often with added lint, to better simulate real wear conditions and typically generates a different pattern and intensity of fuzzing and pilling. The brush pilling method [23] uses a two-stage brushing and rubbing action to increase the amount of protruding fibers on the fabric face and promote their entanglement into pills. The elastomeric pad method [24] uses repeated rubbing against an elastomeric surface to simulate wear conditions and promote the development of surface fuzzing and pilling. The size of the tested specimen depends on the method used to induce surface damage: 125 × 125 mm for the rotating box method; circular specimens 140 mm or 38 mm in diameter for the Martindale method (depending on the standard); square specimens about 100 × 100 mm for the random tumble method; circular specimens 38 mm in diameter for the elastomeric pad method; and either circular specimens 175 mm in diameter or square specimens 320 × 320 mm for the brush pilling method [18,19,20,21,22,23,24].

Observers visually assess abraded fabric specimens under defined conditions [17,19,21,23,24]. According to the EN ISO standard [17], both fuzzing and pilling must be assessed, while ASTM standards [19,21,23,24] require assessment only of the observed occurrence, primarily pilling. The assessment involves visually comparing abraded and non-abraded fabric specimens and/or photographic standards (photographic standards are available for pilling but not for fuzzing). The standard specifies assessment conditions: a distance of 30 to 50 cm from the observer to the specimen surface, a viewing angle of 90° ± 10°, and a specimen surface illumination angle of 5° to 15°, using a D65 artificial daylight source. The result of the visual assessment is a numerical grade from 5 (no change) to 1 (severe fuzzing and/or pilling). According to the EN ISO standard [17], separate grades are required for fuzzing and pilling, while ASTM standards require a single grade, primarily for pilling [19,21,23,24]. Researchers note that limitations of the visual method include observer subjectivity, varying experience, insufficient photographic standards, and the simultaneous occurrence of these phenomena, which makes separate analysis difficult [29,30].

To achieve more objective and reliable grading of fuzzing and pilling, the assessment process is often conducted using instrumental methods, which are permitted but not specifically defined in the standard method [17,31,32]. Instrumental methods typically use digital imaging to capture images of the textile material’s surface, followed by digital image analysis that provides quantitative numerical values for the observed surface changes [33]. Grading with instrumental methods relies on these quantitative values obtained through digital image analysis software. However, these values are not standardized and may vary depending on the specific digital image analysis method, software algorithm, and reference database used, resulting in different metrics such as indexes, percentages, or square units [34].

**Figure 2:**
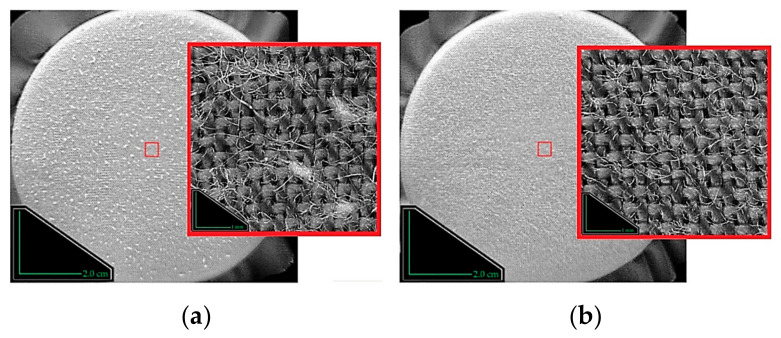
Digital images of the woven fabric surface abraded by the Martindale method: (**a**) occurrence of pilling; (**b**) occurrence of fuzzing.

The literature review indicates that pilling is the most commonly detected form of fabric surface damage using instrumental methods [2,15,29,30,31,32,33,34,35,36,37]. This is mainly because pills are much larger than individual protruding fibers and cast a more noticeable shadow under standard illumination (Figure 2a), resulting in a stronger visual impact that can be reliably captured by surface imaging and quantified through digital image analysis [36,38]. Surface imaging of pilling has been performed using various devices, such as cameras [32,36], scanners [37,39], and microscopes [2,31], which, according to their operating principles, determine the size of the observed area and the specimen’s position during imaging [5]. In contrast, assessing fuzzing with instrumental methods is much more challenging and is rarely reported. This is because the individual protruding fibers of fuzzing, under standard conditions (Figure 2b), cause diffuse light scattering across the textile surface, which appears as a reduction in smoothness or luster and does not create a distinct or strong visual impact [40].

In the rare cases where instrumental methods and digital image processing were used to assess fuzzing, researchers employed specific imaging devices placed directly above the analyzed fabric surface, such as optical microscopes [2,31] and optical coherence tomography (OCT) systems [37,39]. These devices enabled more detailed surface imaging but limited the analyzed specimen’s surface area. In Zhi, C. et al. [31], researchers acquired sequential multifocal images (surface area of 1.73 mm × 1.30 mm; 800 × 600 pixels) of the textile surface using an automatic microscope (M318, BEION Co., Ltd., Shanghai, China) by moving the objective stage along the *z*-axis. Fuzzing occurrence was graded based on the extracted protruding fiber regions obtained from the difference between the depth image and the fitted surface plane. In Sekulska–Nalewajko, J. et al. [37], researchers analyzed surface fuzzing by acquiring 3D volumetric images (0.5 × 0.5 × 0.4 cm; 512 × 512 × 640 voxels) of fabric surfaces using optical coherence tomography with the Spark OCT 1300 tomographic laboratory system (Wasatch Photonics Inc., Morrisville, NC, USA). Fabric fuzzing intensity was quantified and graded using a numerical parameter derived from the protruding fiber volume fraction within the pilling layer and the extracted Haralick texture features. As shown, although the numerical values used to detect the intensity of fabric surface fuzzing in this research differ, the final description of fuzzing intensity was still quantified as a standard grade. In each study, fuzzing was induced using a single standard abrasion method (GB/T 4802.1-2008 in Zhi, C. et al. [31]; EN ISO 12945-2:2020 in Sekulska–Nalewajko, J. et al. [37]), but with multiple types of abradant materials (standard wool abradant fabric, polyamide 6.6 brush, unglazed ceramic plate). Researchers used this approach to address the limitations of a single abrasion method, as abrasion mechanics strongly influence the contact interactions between the specimen surface and the abradant, as well as the spatial distribution and concentration of the induced fuzzing [41,42].

**Figure 3:**
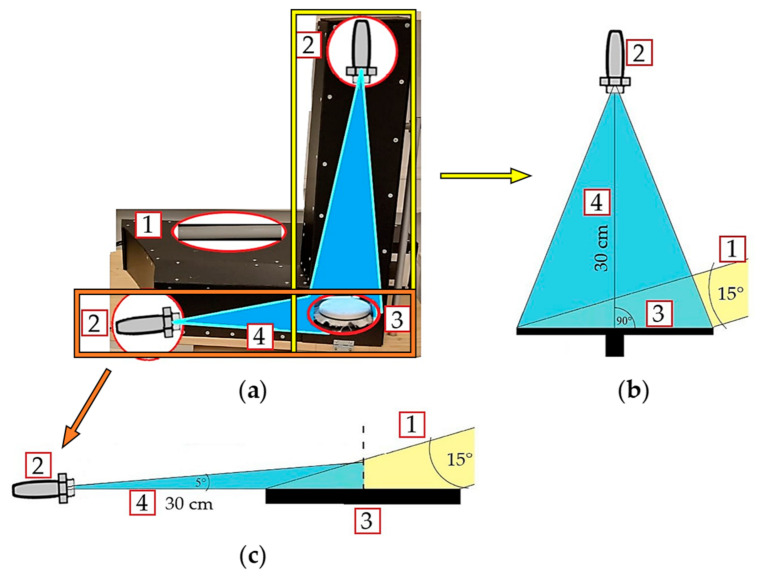
Designed and constructed (**a**) innovative apparatus for advanced digital imaging of fabric specimen surfaces: 1—illumination source position, 2—two camera placement positions and their fields of view, 3—fabric specimen position, 4—distance and angle between the camera and fabric specimen surface; (**b**) first camera position according to standard visual method conditions [17]; (**c**) second camera position with a refined specimen viewing angle.

Based on the literature review, the main limitation of these instrumental methods for analyzing fabric surface pilling and fuzzing is the very small fabric specimen surface area captured by the imaging devices. An innovative approach to textile pilling assessment using uniform digital imaging was introduced through the use of innovative apparatus (Figure 3a) and newly developed image analysis software, previously applied in research for assessing fabric surface pilling [35].

The apparatus features a single specimen holder and two distinct camera mounting positions. The first camera, set at a 90° viewing angle, was used to image the specimen surface to obtain pilling results (Figure 3b), with an artificial daylight (D65) light source. Digital analysis of the images captured by the first camera enabled uniform imaging of the entire fabric specimen surface, fully consistent with the conditions of the standard visual assessment method [17] (Figure 3b). The images produced with this apparatus closely match human visual perception and conventional visual assessment of pilling, as confirmed by very strong Pearson correlation coefficients and very high coefficients of determination in previous research [35].

Given the insufficiently explored possibilities for analyzing surface fuzzing in textile materials, the novelty of this research lies in the advanced digital imaging assessment method that uses the second camera mounting position (Figure 3c) of an innovative apparatus for imaging fabric specimen surface fuzzing. The second camera provides an improved lateral viewing angle of the same fabric specimen under test, enabling advanced digital imaging that makes the visual impact of fuzzing on the fabric surface more distinct from pilling and covers a larger area. This distinguishes the present research, as it enables the innovative apparatus to capture digital images of fabric specimen surfaces from an improved lateral viewing angle, where the visual impact of fuzzing is more distinct from pilling. Therefore, a quantitative assessment of fuzzing on these types of images had not been performed previously [35] and requires a different image analysis method. To address this, a newly developed software tool was designed and implemented to quantify the occurrence and intensity of fuzzing based on height measurements.

In this research, the occurrence of fuzzing was evaluated on six reference single-component, plain-weave woven fabrics made from ring-spun yarns of different polymer-based fibers. To analyze the mechanisms of surface damage formation and compare the intensity of fuzzing results obtained by different methods, surface fuzzing was induced on fabric specimens using an ICI tester (EN ISO 12945-1:2020 [22]) and a Martindale tester (EN ISO 12945-2:2020 [18]), with the number of box revolutions and fuzzing rubs ranging from 125 to 30,000. Both methods induce fuzzing through their distinct abrasion mechanisms and abradant materials, but using the same number of box revolutions and fuzzing rubs makes the specimens comparable. This comparability could not be achieved if fuzzing were induced by care processes, due to the variety of fabric fiber compositions and the different procedures and conditions of their recommended care. To confirm the applicability of the innovative apparatus for evaluating fuzzing results, fuzzing was assessed using both the standard visual method under standard conditions (EN ISO 12945-4:2020 [17]) on the entire woven fabric specimen surface and the newly developed advanced digital imaging assessment method on the lateral side view of the same woven fabric specimen surface, following established grading classes based on the percentage change in fuzzing height.

## 2. Materials and Methods

### 2.1. Textile Materials Used

Six reference single-component plain-weave woven fabrics, made from ring-spun yarns of staple cotton, wool, viscose, polyamide 6.6, polyester, and acrylic fibers, were purchased from SDC Enterprises Limited, Holmfirth, UK, an authorized manufacturer of reference materials for textile testing, and selected for this research. The well-aligned, compact structure of ring-spun yarns, combined with the tightly structured plain-weave fabric, does not promote easy fiber wear. Instead, the yarns are firmly locked within the fabric structure, allowing wear to be distributed more evenly across all yarns [13]. Therefore, this selection was made to achieve better control and to detect a more gradual development of surface damage during the abrasion process. The fabrics and their constituent yarns are light-colored and free from chemically damaged fibers, finishing agent residues, dyes, or fluorescent brightening agents, as they are produced in accordance with ISO 105–F01, F02, F03, F04, and F05 standards [43,44,45,46,47], which serve as process validation tests. The structural properties of the fabrics and the yarn construction used in their production, determined under standard atmospheric conditions (EN ISO 139:2005/A1:2011 [48]; 20 ± 2 °C temperature and 65 ± 4% relative humidity) using standard test methods, are shown in Table 1 and Table 2.

The differences in warp and weft yarn construction (Table 2), despite the identical plain-weave structure and ring-spun yarn spinning technique, introduce structural variation among the selected fabrics. This variation makes the fabrics suitable for this research, as it increases the variability of fabric surface hairiness and helps determine the applicability of the advanced digital imaging assessment method.

### 2.2. Textile Materials Surface Fuzzing Testing Methodology

**Figure 4:**
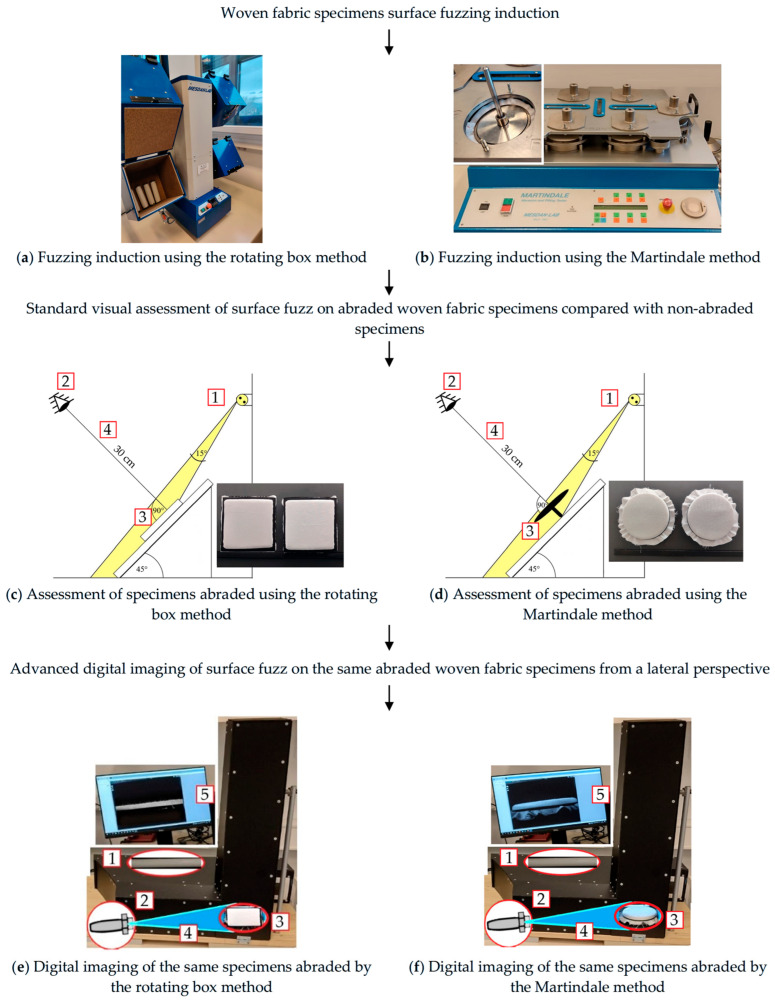
Schematic diagram of the research route for surface fuzzing in textile materials: (**a**,**b**) fuzzing induction on woven fabric specimens using the rotating box and Martindale methods; (**c**,**d**) standard visual assessment [17] of surface fuzz on abraded fabric specimens compared with non-abraded controls; (**e**,**f**) advanced digital imaging of surface fuzz on the same abraded fabric specimens from a lateral perspective: 1—illumination source position, 2—camera placement and field of view, 3—fabric specimen position, 4—distance and angle between the camera and fabric specimen surface, 5—lateral view of the fabric specimen surface.

Figure 4 presents a schematic diagram of the research route for this study, illustrating the fabric specimen preparation process for the advanced digital imaging assessment method of surface fuzzing. The preparation process includes two standard abrasion methods (see Section 2.2.1) to induce fuzzing, followed by the standard visual assessment method (see Section Standard Visual Assessment Method) to grade the visual impact of the induced fuzzing, and the advanced digital imaging method (see Section Advanced Digital Imaging Assessment Method) applied to the same abraded woven fabric specimens to capture digital images in which the visual impact of surface fuzzing is more distinct than that of pilling.

The assessment of surface fuzzing on captured digital images involved fewer digital image processing steps compared to previously reported research [2,31,37,39] or other procedures typically used for pilling assessment [2,15,29,30,31,32,33,34,35,36,37]. Specifically, it did not require complex transformations (such as Fourier and wavelet transforms) or morphological methods to reduce the influence of the fabric’s inherent features, including fiber content [2], fabric structure (weave type, orientation, uniformity, or periodic patterns) [11], color [36], and surface finishes [12]. This simplification is a substantial advantage of the applied approach, achieved primarily by the improved lateral view of the specimen surface, which allows clearer separation between the fuzzing layer and the fabric layer. The quantitative values related to surface fuzzing were determined using newly developed software that operates based on a statistical method [2,34]. The grading of fuzzing intensity by the advanced method is based on the percentage change between the quantitative values determined on abraded and non-abraded fabric specimens.

#### 2.2.1. Textile Materials Surface Fuzzing Induction Methods

Two standard methods were used to induce surface fuzzing in woven fabric specimens. The rotating box method, EN ISO 12945-1:2020 [22], was performed using the ICI tester with four boxes (catalog number 279G, Mesdan S.p.A., Raffa, Italy; Figure 4a), and the Martindale method, EN ISO 12945-2:2020 [18], was performed using the Martindale tester (catalog number 2561E, Mesdan S.p.A., Raffa, Italy; Figure 4b). Both methods were conducted under standard atmospheric conditions (EN ISO 139:2005/A1:2011 [48]).

The rotating box and Martindale methods were selected because their distinctly different abrasion mechanisms produce different types of surface damage. This approach addresses the limitations of using a single abrasion method, as abrasion mechanics significantly affect the contact interactions between the specimen surface and the abradant, as well as the spatial distribution and concentration of the induced fuzzing [41,42]. In the rotating box method [22], square fabric specimens measuring 125 × 125 mm are mounted on polyurethane tubes (product code 6302, batch PT10, SDC Enterprises Limited, Holmfirth, UK) that are 140 ± 1 mm long, 31.5 ± 1.0 mm in outside diameter, and have a mass of 52.25 ± 1.00 g. During abrasion testing, four mounted specimens from the same fabric, sewn in the warp direction, are placed together and simultaneously tumbled at a constant speed of 60 revolutions per minute in a single cork-lined rotating box. Surface damage, i.e., fuzzing on the fabric specimens in this method, results from random abrasion between the four specimens and against the cork-lined interior of the rotating box. In the Martindale method [18], circular fabric specimens with a diameter of 140 ± 5 mm are mounted on a movable test specimen holder over a 90 mm diameter felt underlay (product code 2010, batch WF42, SDC Enterprises Limited, Holmfirth, UK).

The specimens are rubbed against abradant material (diameter 140 ± 5 mm) made from the same fabric as the one being tested and placed on the Martindale tester table, also underlaid with felt (diameter 140 ± 5 mm). Abrasion in the Martindale method is performed under controlled conditions (Lissajous curve motion) and a constant weight of 415 g. Surface damage and the occurrence of fuzzing on fabric specimens in the Martindale method result from controlled and constant abrasion between the fabric specimen and the abradant fabric.

In both standard methods [18,22], the number of control points at which the degree of induced fuzzing should be assessed is specified. In the ICI rotating box method, one control point is defined based on the number of box revolutions (from 14,400 to 18,000). In the Martindale method, six control points are defined (at 125, 500, 1000, 2000, 5000, and 7000), based on the number of fuzzing rubs. Therefore, in this research, a total of twelve control points were defined for both methods (125, 500, 1000, 2000, 5000, 7000, 10,000, 14,000, 18,000, 22,000, 26,000, and 30,000). For each control point, one specimen of each tested fabric was prepared. The comparability of the specimens subjected to the two abrasion methods was experimentally verified by determining a similar duration for a single rotating box revolution (1.0 s) and a single fuzzing rub (1.1 s).

#### 2.2.2. Textile Materials Surface Fuzzing Assessment Methods

##### Standard Visual Assessment Method

The induced occurrence of fuzzing on woven fabric specimens was first evaluated using the standard visual assessment method EN ISO 12945-4:2020 [17]. The assessment took place in a darkened room, where the specimens were placed on a viewing table at a 45° angle and illuminated by artificial daylight (D65) at a 15° angle (Figure 4c,d), within a custom-designed viewing unit. To ensure consistent visual assessment of specimens abraded by both the rotating box method [22] and the Martindale method [18], a custom-made test specimen holder with a felt underlay was designed and constructed (Figure 4c) for the specimens abraded by the rotating box method. This was necessary because these specimens are removed from the polyurethane tubes after the fuzzing induction process.

Three observers graded the fuzzing intensity by visually assessing the surface of the abraded fabric specimens (Figure 4c,d, right position) placed on specimen holders, from a defined distance and angle, and comparing them to the non-abraded fabric specimen (Figure 4c,d, left position). The fuzzing intensity grades range from 5 to 1. The standard defines the grades as follows: 5—no change; 4—slight surface fuzzing; 3—moderate surface fuzzing; 2—distinct surface fuzzing; and 1—dense surface fuzzing [17]. The final grade for each fabric specimen was calculated as the average of the grades assigned by the three observers. If the observers’ grades fell between two consecutive grades, a half grade, expressed as a decimal (e.g., “x.0” or “x.5”), was assigned, and inter-observer variability was reported. This method increased the number of possible grades from five to nine: 1.0, 1.5, 2.0, 2.5, 3.0, 3.5, 4.0, 4.5, and 5.0.

##### Advanced Digital Imaging Assessment Method

**Figure 5:**
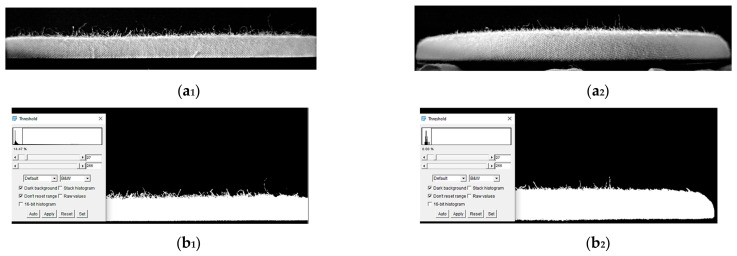

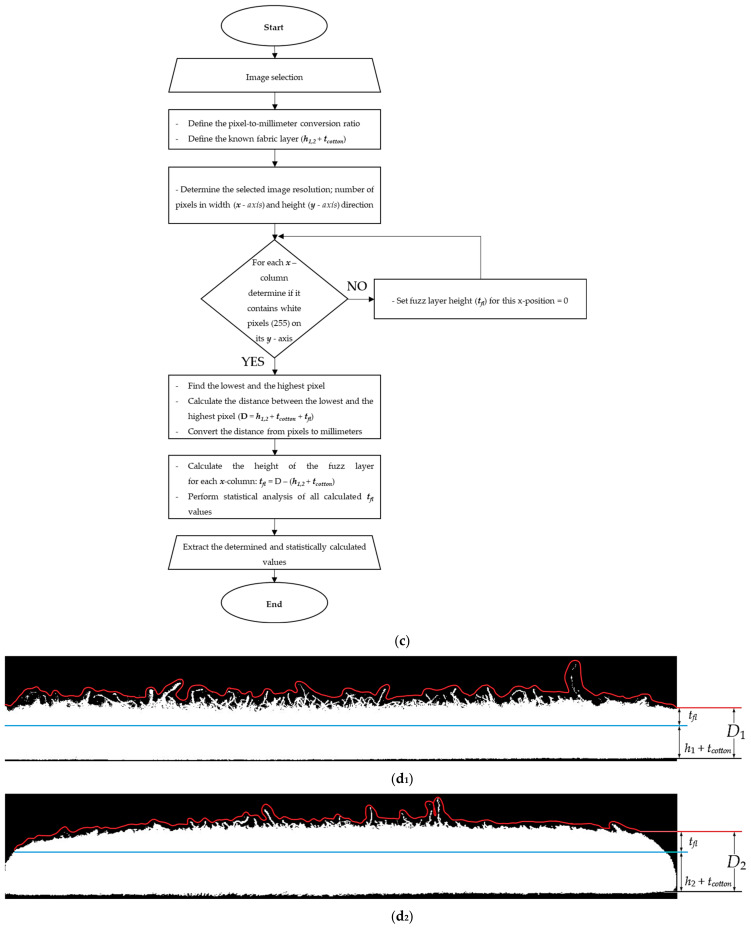
Advanced digital image assessment method—image analysis process: grayscale digital image of the abraded cotton fabric specimen surface after (**a_1_**) 125 box revolutions and (**a_2_**) fuzzing rubs; threshold-adjusted (black and white) images of specimens abraded by (**b_1_**) the rotating box method and (**b_2_**) the Martindale method; (**c**) fuzzing layer analysis using newly developed software; Black and white digital images of fabric specimens abraded by (**d_1_**) the ICI rotating box method and (**d_2_**) the Martindale method, with a blue imaginary interface line between the fabric layer (fabric thickness, *t_cotton_*, and the distance, *h*_1,2_, from the visible side edge of the specimen holders) and the fuzzing layer (*t_fl_*), whose end is marked by a red line.

Digital imaging of the visually assessed fabric specimens was performed using the advanced digital imaging method, specifically the second camera mounting position of the designed and constructed closed-housing innovative apparatus (Figure 3c and Figure 4e,f), which was not used in previous research [35]. The second camera mounting position (Figure 3c) provided an improved lateral view of the specimen at a 5° angle (instead of the conventional 90°), while maintaining the standard distance of 30 cm from the specimen surface, which was illuminated with artificial daylight (D65) at a 15° angle. This perspective was chosen to address the limitations of imaging methods used in previous studies by capturing images in which fuzzing produces a more pronounced visual effect than pilling across a larger surface area of the specimen, distinguishing the present apparatus and digital imaging method from previously reported solutions [2,31,37,39]. The captured images displayed the full side view of the fabric specimen width (10 cm for rotating box specimens and 9 cm for Martindale tester specimens). The digital imaging device used in the apparatus was a Dino-Lite digital microscope camera, catalog number AM-413ZT (AnMo Electronics Corporation, New Taipei City, Taiwan), connected to a computer, where DinoCapture 2.0 software [56] was used to capture and save grayscale digital images at a resolution of 1280 × 1024 pixels. The relationship between pixel values and physical millimeters was established through a scaling procedure using the Dino-Lite calibration sample [57]. Fiji software (version 2.17.0) [58] was used to quantitatively analyze surface fuzzing on the captured grayscale digital images (Figure 5(a_1_,a_2_)) with the advanced digital imaging method.

**Figure 6:**
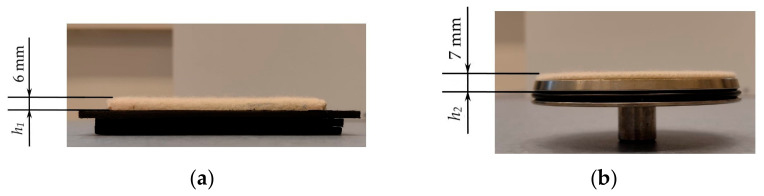
Definition of the imaginary interface line between the fabric and the fuzzing layer: (**a**) distance (*h*_1_) from the visible side edge of the ICI rotating box fabric specimen holder to the top of the holder with felt underlay; (**b**) distance (*h*_2_) from the visible side edge of the Martindale method fabric specimen holder to the top of the holder with felt underlay.

The analysis of digital images begins by setting their threshold value (Figure 5(b_1_,b_2_)). A uniform threshold value of 27 was selected for each of the six fabric samples after a sensitivity analysis, as noticeable variations in fabric surface texture, caused by differences in yarn properties (Table 1 and Table 2), were observed. The analysis involved visually inspecting 18 images of unrubbed specimens (three from each fabric sample) and all specimens abraded using both the rotating box method [22] and the Martindale method [18]. The image analysis method in this research was applied to light-colored samples and does not use any previous dataset. Therefore, before applying this method to other fabrics, the threshold parameters must be recalibrated, as different fiber types, fabric structures, colors, and surface finishes can influence the results. The digital images with the adjusted threshold value were then converted to a black-and-white (binary) version containing only two pixel values: 0 for the background (black) and 255 for the surface hairiness of non-abraded fabric specimens or the fuzzing layer of abraded fabric specimens (white). The next step of the image analysis was performed using a newly developed software tool (Figure 5c) written as an ImageJ macro (.ijm file) for execution within the Fiji (Laboratory for Optical and Computational Instrumentation and University of Wisconsin-Madison, Madison, United States) (ImageJ-based v1.8.0) software. The macro systematically processes the binarized image column by column across its full width of 1280 pixels (*x*-direction). For each pixel column, the software locates the highest and lowest white pixel along the vertical axis (*y*-direction) and calculates the vertical distance between these two positions (*D*, Equation (1)). This distance includes not only the hairiness or fuzzing layer but also the distance from the visible side edge of the specimen holder to the top of the holder with felt underlay (Figure 6a,b), and the fabric layer, so an additional step was added. In this step, an imaginary interface line between the fabric layer and the surface hairiness layer (for non-abraded fabric specimens) or the fuzzing layer (for abraded fabric specimens), was introduced. The position of the interface line was defined as the sum of two experimentally determined values: first, the distance from the visible side edge of the specimen holder to its top (*h*_1_ for the ICI rotating box specimen holder; *h*_2_ for the Martindale method specimen holder), with felt underlay (Figure 6a,b); and second, the thickness (*t_m_*) of the non-abraded fabric determined by the standard method [50], as shown in Table 1. The interface line enabled quantification of surface fuzzing intensity as the height of the fuzzing layer (*t_fl_*, mm), which is characteristic for each fabric sample.

*D* = *h*_1,2_ + *t_m_* + *t_fl_* (1)

where:

- *D*—distance between the highest and lowest white pixel along the vertical axis (*y*-direction);
- *h*_1,2_—distance from the visible side edge of the fabric specimen holder to the top of the holder with felt underlay (*h*_1_ for the ICI rotating box fabric specimen holder; *h*_2_ for the Martindale method fabric specimen holder);
- *t_m_*—fabric layer = fabric thickness;
- *t_fl_*—hairiness or fuzzing layer; height of the hairiness (non-abraded) or fuzzing (abraded) layer on the fabric specimen’s surface.

Therefore, the determined distances for each individual pixel column (*D*) are reduced by the *h*_1_ or *h*_2_ + *t_m_* values, isolating the net height mainly attributable to the fabric specimen’s surface hairiness (non-abraded) or fuzzing (abraded) layer (Figure 5(d_1_,d_2_)). This process is performed independently for each of the 1280 image pixel columns, resulting in 1280 discrete measurements of the surface fuzzing layer height for each abraded fabric specimen and the surface hairiness layer height for each non-abraded fabric specimen.

Based on the 1280 values of the fuzzing layer height (*t_fl_*), the following quantitative statistical parameters were calculated: the arithmetic mean ($\bar{t_{fl}}$) with its corresponding coefficient of variation, and the median ($\tilde{t_{fl}}$). The median change (∆*t_fl_*) of the fuzzing layer height—or, more precisely, its percentage change (∆$\tilde{t_{fl}}$)—was used to define the nine grading classes, as shown in Table 3. The median was chosen because it is not influenced by extremely high or low values, which are common across the fabric specimen surface due to non-uniform fiber protrusion and accumulation during abrasion. Therefore, the percentage change based on the median of the fuzzing layer height provides a more objective, robust, and consistent measure of overall fuzzing behavior compared to parameters affected by these outliers.

(2) $$∆\tilde{t_{fln}}=\frac{\left(\tilde{t_{fln}})\times (\tilde{t_{fl0}}\right)}{\left(\tilde{t_{fl0}}\right)}\times 100$$

where

- ∆$\tilde{t_{fln}}$—percentage change in the median fuzzing layer height;
- $\tilde{t_{fln}}$—median fuzzing layer height of the abraded fabric specimen;
- $\tilde{t_{fl0}}$—median hairiness layer height of the non-abraded fabric specimen;
- n—number of control points (number of ICI box revolutions or Martindale fuzzing rubs).

## 3. Results and Discussion

The results first present and discuss the fuzz grades assigned using the standard visual method. Next, the results obtained with the proposed advanced digital imaging assessment method are provided. These include cross-sectional elemental mapping grayscale digital images of fabric specimens under different degrees of abrasion, captured by innovative apparatus at each of twelve control points, as well as quantitative values of statistical parameters for fuzzing layer height, determined by advanced digital image analysis software: the percentage change in median fuzzing layer height and the corresponding fuzz grades assigned according to the defined nine fuzz grading classes.

### 3.1. Fuzz Grades Assigned by Standard Visual Assessment Method

Fuzz grades assigned by three observers, along with inter-observer variability values where applicable, using the standard visual assessment method for each fabric specimen abraded by the rotating box method [22] (M1) and the Martindale method [18] (M2) after each of twelve predefined control points (125, 500, 1000, 2000, 5000, 7000, 10,000, 14,000, 18,000, 22,000, 26,000, and 30,000 box revolutions or fuzzing rubs), are shown in Table 4.

Lower surface fuzzing intensity was observed on fabric specimens abraded by the Martindale method [18] (method M2), while a more pronounced and significantly more persistent occurrence of surface fuzzing was observed on fabric specimens abraded by the rotating box method [22] (method M1). This difference is attributed to the distinct abrasion mechanisms of the two methods. In the Martindale method, controlled and constant abrasion contact between the fabric specimen and the abradant fabric causes more severe abrasion, leading to faster breakage and removal of surface-protruding fiber ends, that is, higher fiber shedding. In the rotating box method (M1), the uneven and inconsistent abrasion contact between the specimen and the cork-lined interior of the rotating box [41,42] causes milder abrasion, resulting in a greater number of protruding fiber ends remaining anchored to the fabric structure for a longer period before their detachment or breakage. Although the abrasion mechanism significantly influences the intensity and persistence of fuzzing, in the tightly woven plain weave fabrics tested, the dominant factor governing fuzzing intensity and fiber shedding remains the type of polymer-based fiber from which the fabric is made and its properties, particularly tensile strength, bending rigidity, surface morphology, and inter-fiber cohesion within the ring-spun yarn structure [2,9,10].

Cotton and acrylic fabric specimens abraded using the M1 method showed similar, predominantly moderate surface fuzzing intensity (grades between 3.0 and 4.0), which persisted across all control points (from 125 to 30,000 rotating box revolutions). During abrasion with the M2 method, the fuzzing behavior of these fabrics differed significantly. Cotton specimens abraded by M2 exhibited very pronounced fuzzing intensity at the first two control points (grade 1.0 after 125 fuzzing rubs; grade 3.0 after 500 fuzzing rubs), after which the protruding fibers quickly became entangled and formed pills [35]. At higher numbers of fuzzing rubs, the occurrence of pilling and fuzzing on cotton fabric specimens rapidly diminished. This indicates that cotton fabrics are sensitive to abrasion due to the moderate tensile strength and relatively low wear resistance of cotton fibers. Initially, this leads to significant surface fuzzing and pilling, but with prolonged abrasion, these pills and loose fibers detach rapidly, resulting in a smoother surface appearance of the specimens [3,35]. On acrylic fabric specimens abraded with method M2, only slight fuzzing (grade 4.0) was observed at lower numbers of fuzzing rubs (from 125 to 7000). This can be attributed to the wool-like crimp and moderate tensile strength of acrylic fibers, which facilitate the formation of protruding fibers at lower numbers of fuzzing rubs. However, after longer abrasion periods, their relatively lower tensile strength leads to gradual fiber breakage and detachment [8].

Wool fabric specimens abraded by the M1 method exhibited surface fuzzing between 2000 and 14,000 revolutions (grades 2.0 to 3.0), while specimens abraded by the M2 method showed an earlier onset (grade 1.0 after 125 fuzzing rubs) and earlier disappearance (grade 4.0 after 5000 fuzzing rubs). Pilling was slight (few pills) and occurred together with fuzzing [35]. Surface fuzzing on wool fabrics is primarily attributed to the natural crimp, scale structure, and variable fiber length of wool fibers, which facilitate easy protrusion from the fabric structure. Although inter-fiber friction provides cohesion within the yarns, these properties promote pronounced fiber protrusion. This explains why fuzzing on wool fabrics persisted much longer under the milder, random abrasion of the M1 method compared to the more intense and continuous abrasion of the M2 method [4].

Polyester fabric specimens generally showed a very low tendency for surface fuzzing. Specimens abraded by the M1 method exhibited only a slight degree of fuzzing between 2000 and 10,000 revolutions (predominantly grade 3.0), while no fuzzing was observed on specimens abraded by the M2 method (all grade 5.0). However, both M1 and M2 fabric specimens developed pilling after prolonged abrasion. This behavior is primarily attributed to the smooth surface morphology and high tensile strength of polyester fibers, which provide strong inter-fiber cohesion and significantly limit the initial protrusion of fiber ends from the fabric structure. Although minimal fuzzing can still occur under milder abrasion conditions (M1), any protruding fibers are quickly broken or re-embedded due to the structural stability of the fabric. This explains why detectable fuzzing was observed only under the milder, random abrasion of the M1 method, while the more intense and continuous abrasion of the M2 method completely prevented it. Nevertheless, prolonged abrasion eventually leads to pilling in both methods [7].

Viscose and polyamide 6.6 fabric specimens exhibited a greater tendency for surface fuzzing when abraded using the M1 method. In viscose fabric specimens, high fuzzing intensity (grades between 1.0 and 2.0) was observed at lower numbers of box revolutions (125 to 7000), after which the intensity gradually decreased (grades between 3.0 and 5.0). In polyamide 6.6 fabric specimens, high fuzzing intensity (predominantly grade 1.0) was observed at each of the twelve control points (125 to 30,000 box revolutions). For viscose and polyamide 6.6 fabric specimens abraded by the M2 method, a high tendency for surface pilling was detected. This was observed in both fabric types, even after a very low number of fuzzing rubs (from 125). Pilling formation in both fabrics led to lower fuzzing, as the free protruding fiber ends were quickly entangled into pills. The pilling formation rate for viscose fabric specimens was more sensitive to abrasion time and, at higher numbers of fuzzing rubs, showed a higher rate of fuzzing and pilling detachment. In contrast, polyamide 6.6 fabric specimens showed only a gradual increase in the number of pills. The high and early-occurring fuzzing intensity in viscose fabrics is primarily due to the low bending rigidity and reduced inter-fiber cohesion of viscose fibers, which enable rapid release and protrusion of fiber ends from the fabric structure. However, because of their relatively low abrasion resistance, these protruding fibers are progressively broken and removed after longer abrasion periods, resulting in a gradual reduction in both fuzzing and pilling with prolonged abrasion [5]. In contrast, the persistent and pronounced fuzzing observed on polyamide 6.6 fabrics, especially under the milder random abrasion of the M1 method, can be attributed to the high tensile strength and excellent wear resistance of polyamide 6.6 fibers. These properties allow protruding fiber ends to remain firmly anchored within the fabric structure for a prolonged period without breaking, even after extensive abrasion during the M2 abrasion method [6].

From the observed changes, it is evident that reduced excessive fiber detachment and more evenly distributed abrasive forces—resulting from the tight plain weave structure of the tested fabrics and the compact yarn structure of the ring-spun yarns—enabled consistent monitoring and tracking of fuzzing occurrence at all twelve predefined control points [11,13]. The influence of fiber type and the different abrasion methods was clearly observed. Finally, it should be noted that visual assessment does not allow for a clear distinction between the mechanisms of surface damage during abrasion—whether additional protruding fibers are being pulled out or wear has occurred, resulting in the shortening of the fabric’s natural surface hairiness.

### 3.2. Results Obtained Using the Advanced Digital Imaging Assessment Method

#### 3.2.1. Quantitative Statistical Parameters of Fuzzing Intensity

Quantitative statistical parameters of fuzzing intensity were obtained from captured digital images. Table 5 presents these images as cross-sectional elemental mappings of grayscale side-view digital images of fabric specimens placed on specimen holders for the ICI rotation box method (M1) and the Martindale method (M2). The images are taken from the central areas (width of 100 px) of non-abraded (0) and each of the twelve abraded fabric specimens (ranging from 125 to 30,000 box revolutions or fuzzing rubs), allowing observation of the development of fuzzing and the bonding state between protruding fibers under varying degrees of abrasion. The images clearly show differences in the intensity and dynamics of surface damage to abraded fabric specimens, which directly depend on the abrasion method used and the type of fabric tested. Quantitative numerical values for the non-abraded fabric surface hairiness layer and the abraded fabric specimens’ fuzzing layer height, determined by the advanced digital imaging assessment method, include the arithmetic mean ($\bar{t_{fl}}$), the corresponding coefficient of variation (*V*), the median ($\tilde{t_{fl}}$), and the percentage change (∆$\tilde{t_{fl}}$) of the median fuzzing layer height, as shown in Table 6, Table 7 and Table 8. These values are obtained from grayscale digital images captured at a refined viewing angle using the innovative apparatus (Figure 4e,f) and newly developed software (see Section Advanced Digital Imaging Assessment Method).

The initial surface hairiness layer heights of the non-abraded fabrics, based on the arithmetic mean of surface hairiness values for fabric specimens at the 0–control point ($\bar{t_{fl}}$), varied by fabric type (Table 6). For example, non-abraded cotton fabric specimens have the highest surface hairiness layer, while polyester and acrylic have the lowest, with high coefficients of variation (ranging from 38% to 56%). This indicates that the parameters used in the newly developed software for the advanced digital imaging assessment method (see Section Advanced Digital Imaging Assessment Method) were well chosen, as they effectively captured height oscillations within the surface hairiness layer. Height oscillations within the fabric surface hairiness layer, variations in the shape of specimen holders for the ICI rotation box method (M1) and the Martindale method (M2) shown in Figure 5(d_1_,d_2_), and a small difference of one centimeter in fabric specimen width all affect the consistency of hairiness layer height results. However, these factors also demonstrate the method’s applicability to fabric specimens of different sizes.

The measured fuzzing layer heights of the abraded fabric specimens differed from the initial surface hairiness layer heights of the non-abraded fabrics. These changes were strongly influenced by the applied abrasion method. As a progressive wear process, abrasion initially causes surface roughening and the formation of new protruding fibers (fuzzing). With prolonged abrasion, depending on the method used, varying degrees of fiber breakage, fiber detachment, and pilling formation occur [13,14], as shown in the elemental mapping of grayscale digital images of fabric specimens in Table 5.

Cotton fabric specimens showed an initial decrease in fuzzing layer height after the first two control points (125 and 500 control points) under both abrasion methods (1.10 mm for M1 and 1.77 mm for M2). With further abrasion, a gradual increase in fuzzing layer height was observed, accompanied by pilling formation. However, this increase never reached the initial height of the non-abraded fabric specimens. This behavior is consistent with the moderate tensile strength and relatively low abrasion resistance of cotton fibers [3], which allow rapid formation of new protruding fibers while also enabling progressive detachment of existing fibers and pills due to ongoing wear, ultimately resulting in a smoother surface appearance. Wool fabric specimens abraded by the M1 method exhibited a sharp initial drop in fuzzing layer height (down to 0.70 mm after 125 box revolutions), followed by further decline with noticeable oscillations throughout the test. In contrast, the M2 method caused an initial increase in fuzzing layer height, followed by a gradual decrease over longer abrasion periods. These results indicate that random abrasion in the M1 method mainly caused breakage and detachment of already protruding fibers, with limited release of new ones. Conversely, the more intense and controlled abrasion in the M2 method initially promoted the protrusion of new fibers, which were then broken and removed with continued abrasion. Viscose fabric specimens abraded using the M1 method showed an initial increase in fuzzing layer height, reaching 2.45 mm after 125 box revolutions. However, with continued abrasion, pilling occurred, accompanied by noticeable oscillations and a progressive decline in the height of the fuzzing layer. These oscillations and the final decline are evident in the elemental mapping of grayscale digital images of viscose fabric specimens abraded using the M1 method (see Table 5). In contrast, the M2 method caused a steady initial decrease in fuzzing layer height (from 2.09 mm to 1.40 mm), followed by pilling formation. Prolonged abrasion with this method led to more moderate fluctuations and a gradual downward trend in fuzzing layer height. This behavior is primarily due to the low bending rigidity and poor inter-fiber cohesion of viscose fibers [5], which allow rapid initial protrusion. However, because of their relatively low abrasion resistance, prolonged abrasion mainly causes fiber breakage and detachment. Especially under the milder but prolonged action of the M1 method, this results in the breakage of existing protruding fibers without significant release of new ones, causing a substantial reduction in fuzzing layer height. Polyamide 6.6 fabric specimens abraded by the M1 method showed an initial decrease in fuzzing layer height (down to 1.04 mm after 125 box revolutions), followed by a strong upward trend with pronounced oscillations and high peaks (reaching 3.73 mm at 26,000 box revolutions), resulting in a final height of 2.98 mm—nearly double the initial value. In contrast, specimens abraded by the M2 method exhibited only a slight initial decrease (to 1.74 mm) and then remained relatively stable throughout the test (final height 1.89 mm). This distinct behavior is closely related to the high tensile strength and excellent wear resistance of polyamide 6.6 fibers [6]. Under the milder, random abrasion of the M1 method, protruding fiber ends remained anchored for a long time, enabling continuous fuzz accumulation and a progressive increase in fuzzing layer height, with pilling occurring. Under the more intense abrasion of the M2 method, pilling formed at early stages (from 125 rubs), as newly protruding fibers were continuously entangled into new pills, and their number increased with abrasion time. The visual impact of the formed pills is already detectable after only 500 fuzzing rubs, as shown by the elemental mapping of grayscale digital images of polyamide 6.6 fabric specimens abraded by the M2 method. This continuous conversion of protruding fibers into pills prevented further growth of the fuzzing layer height, resulting in the observed stagnation in fuzzing layer height. Polyester fabric specimens abraded by both the M1 and M2 methods showed an increase in fuzzing layer height compared to the initial surface hairiness. The increase was more pronounced with the M2 method, reaching a final height of 1.39 mm after 30,000 cycles, while the M1 method resulted in a more moderate increase (final height 0.94 mm) and stronger oscillations. Pilling occurred during prolonged abrasion in both methods. As with polyamide 6.6, there were no clear signs of wear or significant fiber detachment. Instead, the newly protruding fibers were continuously entangled into pills, whose number increased with abrasion time [35]. This resulted in a specific surface appearance where protruding fibers and formed pills created a dense layer, increasing the overall height of the fuzzing layer. This effect was clearly visible in the elemental mapping of grayscale digital images of polyester fabric specimens after 5000 box revolutions (M1) and fuzzing rubs (M2). Acrylic fabric specimens abraded by both methods showed an increase in fuzzing layer height. Specimens abraded by the M2 method produced a significantly higher and more stable increase, while those abraded by the M1 method exhibited more frequent oscillations, with the height remaining closer to the initial values. No pilling was observed on specimens abraded by either method. This moderate fuzzing behavior is consistent with the wool-like crimp and moderate tensile strength of acrylic fibers, which facilitate the formation of protruding fiber ends, particularly under the more intense and controlled abrasion of the M2 method. The limited increase under the milder M1 method is primarily due to a higher tendency for fiber break-off rather than the release of new protruding fibers from the fabric structure [8].

The coefficients of variation (*V*) for the arithmetic mean of surface fuzzing layer height values of abraded fabric specimens (Table 6) were significantly higher for all tested fabric specimens when abraded using the rotation box method (M1). This is consistent with the nature of the abrasion process in the M1 method, which is random and controlled only by the box rotation speed (60 revolutions per minute), resulting in less uniform and more variable surface damage.

Given the high coefficients of variation, the median value of the surface fuzzing layer height was also calculated to reduce the impact of extreme values and provide a more reliable estimate of the actual fuzzing layer height. As shown in Table 7, the median values are generally lower than the corresponding arithmetic means, particularly for fabric specimens with high coefficients of variation (viscose, wool, cotton, polyester, and acrylic), especially those abraded by the M1 method.

Table 8 shows the percentage change in the median fuzzing layer height. These values represent the percentage differences between the median fuzzing layer heights measured on fabric specimens abraded by each of the twelve predefined numbers of box revolutions or fuzzing rubs (ranging from 125 to 30,000) and the median surface hairiness layer height of non-abraded fabric specimens. Positive percentage change values indicate fuzzing, which refers to the roughening of existing fibers and the emergence of new protruding fibers. Negative percentage change values indicate surface damage and wear, resulting from the breakage or detachment of existing protruding fibers. This presentation of the results makes it easy to identify the type and intensity of surface damage caused by abrasion, whether wear or fuzzing.

Therefore, based on the observed results, the changes are predominantly positive for polyester, acrylic, and polyamide 6.6 fabric specimens, while they are mainly negative for cotton, wool, and viscose fabric specimens. Polyester and acrylic fabric specimens show positive percentage changes due to the smooth surface morphology and strong fiber cohesion of polyester fibers, as well as the moderate tensile strength and wool-like crimp of acrylic fibers, which limit the initial presence of protruding fibers and enable their gradual formation under abrasion [7,8]. Polyamide 6.6 fabric specimens show distinct values when abraded by different methods because of their high tensile strength, wear resistance, and pronounced tendency to form pills [6,35]. Fabric specimens abraded by the Martindale method (M2) exhibit more uniform and sustained formation and entanglement of new protruding fibers into pills, resulting in lower oscillations in fuzzing layer heights, close to the height of the non-abraded fabric specimens’ surface hairiness layer. In contrast, the mild and uneven abrasion caused by the rotating box method (M1) leads to higher values of fuzzing layer height, as the breakage and detachment of protruding fibers is low. Wool, cotton, and viscose fabric specimens generally show negative percentage changes due to their relatively low tensile strength, fiber cohesion, and abrasion resistance, which lead to rapid breakage and detachment of protruding fibers under prolonged abrasion and prevent sustained fuzzing buildup [3,4,5].

#### 3.2.2. Fuzz Grades Assigned by the Advanced Digital Imaging Assessment Method

Table 9 presents the fuzz grades assigned by the advanced digital imaging assessment method. These grades are based on the percentage change in the median fuzz layer height values shown in Table 8 and the nine fuzz grading classes defined in Table 3.

The results show that cotton, wool, and viscose fabric specimens assigned grades of 1.0 to 3.0 by the standard visual assessment method (Table 4) were often graded as 5.0 by the advanced digital assessment method, regardless of whether they were abraded by the rotating box method (M1) or placed on standard Martindale method holders (M2). This difference occurs because observers cannot visually detect wear that causes a slight decrease in the fuzzing layer height compared to the surface hairiness layer of non-abraded fabric specimens. Polyester and acrylic fabric specimens, as indicated by mainly positive percentage changes in median fuzzing layer height (Table 8), tend to exhibit fuzzing. Therefore, when graded by the advanced digital assessment method (Table 9), fabric specimens that received higher grades, such as 4.0 and 5.0, in the standard visual assessment method (Table 4) were often assigned significantly lower grades, such as 1.0. This is due to the minimal visual impact of the low surface hairiness layer height in non-abraded fabric specimens, which led to an underestimation of fuzzing intensity during standard visual assessment. This is especially evident for acrylic fabric specimens abraded by the Martindale method (M2), where the advanced digital assessment method detected more pronounced fuzz development. Polyamide 6.6 fabric specimens abraded by both the M1 and M2 methods generally received higher fuzz grades when assessed by the advanced digital assessment method (mostly 5.0, Table 9). However, lower grades were still observed for fabric specimens abraded by the M1 method after 26,000 and 30,000 box revolutions, indicating that the uneven abrasion of the rotating box method results in a higher occurrence of fuzzing.

**Figure 7:**
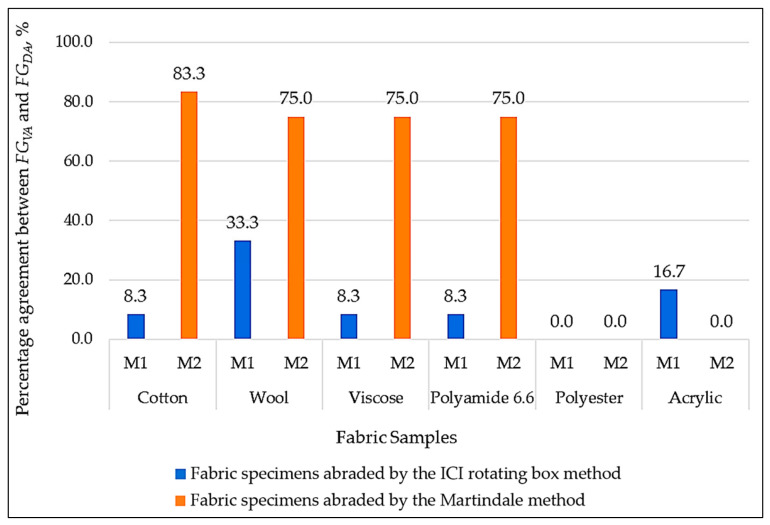
Graphical representation of the percentage agreement between surface fuzzing grades assigned by the standard visual method (*FG_VA_*) and the advanced digital assessment method (*FG_DA_*).

To support this, Figure 7 provides a graphical representation of percentage agreement, illustrating how closely fuzz grades assigned using the advanced digital assessment method (*FG_DA_*) correspond to those assigned by the standard visual method (*FG_VA_*).

Percentage agreement, also known as observed or simple percentage agreement, is a basic measure of concordance between two assessment methods. It is calculated as the number of cases in which both methods assign the same grade, divided by the total number of assessments, and expressed as a percentage [59]. For the rotating box method (M1), agreement values are very low: 0% for polyester fabric, 8.3% for cotton, viscose, and polyamide 6.6 fabrics, 16.7% for acrylic fabric, and 33.3% for wool fabric. The Martindale method (M2) shows significantly higher percentage agreement values: 75.0% for wool, viscose, and polyamide 6.6 fabrics, and 83.3% for cotton fabric, but a notably lower value of 0% for polyester and acrylic. This is due to the low surface hairiness of non-abraded polyester and acrylic fabrics and the dense surface fuzzing, which during visual assessment appears as part of the fabric layer. The *t*-test results (*p*-values) support the trends observed in the percentage agreement analysis (Figure 7). For low agreement values, the *t*-test confirmed that the differences are statistically significant (*p* < 0.05). Conversely, high agreement values indicate that there is no statistically significant difference (*p* ≥ 0.05) between the grades assigned by the two assessment methods (*F_GVA_* and *F_GDA_*) [60].

This confirms that the advanced digital assessment method enables a clearer distinction between the occurrence of fuzzing and wear on the fabric surface, as the visual impact of these changes is strongly influenced by the surface hairiness layer height of non-abraded fabric specimens.

## 4. Conclusions

The aim of this research was to address the challenge of analyzing surface fuzzing on abraded fabric specimens, as its occurrence is often accompanied by pilling, making it difficult to distinguish between the two during the standard visual assessment method EN ISO 12945-4:2020. In this study, fabric specimens from six single-component woven fabrics—cotton, wool, viscose, polyamide 6.6, polyester, and acrylic—were abraded using the rotation box method (EN ISO 12945-1:2020) and the Martindale method (EN ISO 12945-2:2020) for twelve predefined numbers of box revolutions or fuzzing rubs. These specimens were then evaluated using an advanced digital imaging assessment method. The surface of each specimen was captured with an innovatively constructed apparatus, whose refined viewing angle enabled side-view images of the fabric surface, highlighting the visual impact of fuzzing as surface fuzzing layer height.

This approach allowed fuzzing to be observed across the entire width of the fabric specimen and quantified as surface fuzzing layer height using newly developed software within the image analysis program Fiji, which is described in detail in this research. Grading and distinguishing between fuzzing and wear of the fabric surface were achieved by determining the percentage change in the median fuzzing layer height. This made it possible to distinguish the mechanisms of surface damage during abrasion and to determine whether surface fuzzing (protruding fiber formation) or surface damage and shortening of the fabric’s natural surface hairiness (breaking or pulling out of fibers) had occurred. This approach represents a significant advantage over the visual assessment method, which does not allow a clear distinction between the two.

The findings demonstrate the applicability of the proposed advanced digital imaging assessment method, as it successfully quantifies fuzz formation and provides objective insight into the fabric’s propensity for fuzz development and wear resistance. This method can be applied to a wide range of textile materials, including fiber blends, different yarn structures, various fabric constructions, and diverse finishing treatments. Future research will further improve and evaluate the performance of image system and the influence of surface appearance on various textile materials.

## Figures and Tables

**Table 1:** Structural properties of the woven fabrics, including standard deviation values and manufacturer production data.

Fabric Production Data	Mass per Unit Area, gm^−2^ (EN 12127:1997 [49])	Thickness, mm (EN ISO 5084:1996 [50])	Warps, cm^−1^	Wefts, cm^−1^
			(ISO 7211-2:2024 [51])	
100% Cotton, Prod. Code 1520, Batch LB46/2 ISO 105–F02:2009 [43]	114.4 ± 0.87	0.31 ± 0.00	32.0 ± 0.5	37.0 ± 0.5
100% Wool Prod. Code 1920, Batch W28/8 ISO 105–F01:2001 [44]	124.6 ± 0.15	0.35 ± 0.00	22.0 ± 0.5	21.0 ± 0.5
100% Viscose Prod. Code 1820, Batch VR28/45 ISO 105–F02:2009 [43]	141.6 ± 0.73	0.29 ± 0.00	32.0 ± 0.5	23.0 ± 0.5
100% Polyamide 6.6 Prod. Code 1620, Batch N42/29 ISO 105–F03:2001 [45]	131.4 ± 0.11	0.40 ± 0.01	19.0 ± 0.0	21.0 ± 0.5
100% Polyester Prod. Code 1720, Batch P37/42 ISO 105–F04:2001 [46]	134.2 ± 0.48	0.29 ± 0.00	25.0 ± 0.5	18.0 ± 0.0
100% Acrylic Prod. Code 1120, Batch A17/7 ISO 105–F05:2001 [47]	152.1 ± 0.28	0.44 ± 0.00	20.0 ± 0.0	15.0 ± 0.5

**Table 2:** Yarn construction parameters and corresponding standard deviation values, where applicable.

Yarn Construction Parameters		Yarn Direction	Cotton	Wool	Viscose	Polyamide 6.6	Polyester	Acrylic
Twist Direction ISO 2:1973 [52]	Two–Ply Yarn	Warp	-	S	-	S	S	S
		Weft	-	S	-	-	-	S
	Single Spun Yarn	Warp	Z	Z	Z	Z	Z	Z
		Weft	Z	Z	Z	Z	Z	Z
Linear Density ISO 7211-5:2021 [53]	Two–Ply Yarn	Warp	-	25.8 ± 1.32	-	41.5 ± 0.13	40.5 ± 0.51	38.7 ± 0.12
		Weft	-	28.0 ± 0.29	-	-	-	39.5 ± 1.52
	Single Spun Yarn	Warp	13.6 ± 0.12	12.0 ± 0.23	23.3 ± 0.19	20.0 ± 0.16	19.0 ± 0.10	18.0 ± 0.14
		Weft	16.5 ± 0.22	13.0 ± 0.34	25.3 ± 0.30	20.8 ± 0.35	21.7 ± 0.29	28.0 ± 0.43
Twist Number	Two–Ply Yarn	Warp	-	707 ± 98	-	454 ± 37	507 ± 61	650 ± 40
	EN ISO 2061:2015 [54]	Weft	-	750 ± 69	-	-	-	668 ± 38
	Single Spun Yarn	Warp	828 ± 28	861 ± 28	551 ± 18	697 ± 26	689 ± 37	788 ± 33
	ISO 17202:2002 [55]	Weft	893 ± 37	881 ± 32	604 ± 27	678 ± 33	646 ± 26	795 ± 25

**Table 3:** Nine fuzz grading classes created based on the percentage change in the median height of the fuzzing layer (∆$\tilde{t_{fln}}$).

Fuzz Grade	Fuzz Grading Classes Based on the Percentage Change in the Median Height of the Fuzzing Layer
5.0	≤11.11
4.5	from 11.12 to 22.22
4.0	from 22.23 to 33.33
3.5	from 33.34 to 44.44
3.0	from 44.45 to 55.55
2.5	from 55.56 to 66.66
2.0	from 66.67 to 77.77
1.5	from 77.78 to 88.88
1.0	≥88.89

**Table 4:** Fuzz grades assigned by the visual assessment method (*FG_VA_*) for abraded fabric specimens (after 125 to 30,000 control points, corresponding to surface fuzzing), with inter-observer variability values where detected.

Control Points	Fuzz Grades—Standard Visual Assessment Method											
	Cotton		Wool		Viscose		Polyamide 6.6		Polyester		Acrylic	
	M1 *	M2 **	M1 *	M2 **	M1 *	M2 **	M1 *	M2 **	M1 *	M2 **	M1 *	M2 **
125	3.0	1.0	5.0	1.0	1.0	3.0	1.0	4.0	5.0	5.0	4.0	4.0
500	3.0	3.0	5.0	2.0	2.0	3.0	1.0	4.0	4.0	5.0	5.0	4.0
1000	3.0	5.0	5.0	3.0	1.0	5.0	1.0	5.0	5.0	5.0	5.0	4.0
2000	3.0	5.0	3.0	4.0	1.0	5.0	1.0	5.0	3.0	5.0	3.0	4.0
5000	4.0	5.0	3.0	5.0	1.0	5.0	1.0	5.0	3.0	5.0	2.0	4.0
7000	3.0	5.0	2.0	5.0	2.0	5.0	1.0	5.0	4.0	5.0	3.0	4.0
10,000	3.0	5.0	3.0	5.0	4.0	5.0	2.0	5.0	3.0	5.0	4.0	5.0
14,000	4.0	5.0	2.5 ± 0.5	5.0	3.0	4.0	1.0	5.0	5.0	5.0	2.5 ± 0.5	5.0
18,000	4.0	5.0	4.0	5.0	3.0	5.0	1.0	5.0	5.0	5.0	3.0	5.0
22,000	3.0	5.0	4.0	5.0	3.0	5.0	1.0	5.0	3.0	5.0	4.0	5.0
26,000	4.0	5.0	5.0	5.0	5.0	5.0	2.0	5.0	4.0	5.0	3.0	5.0
30,000	5.0	5.0	4.0	5.0	4.0	5.0	1.0	5.0	5.0	5.0	5.0	5.0

* The ICI rotating box method; ** Martindale method.

**Table 5:** Cross-sectional elemental mapping of central areas (width 100 px) from grayscale side-view digital images of non-abraded (0) and each of the twelve abraded fabric specimens (ranging from 125 to 30,000 box revolutions or fuzzing rubs), placed on specimen holders for the ICI rotation box method (M1) and the Martindale method (M2).

Fabric	Abrasion Method	Elemental Mapping of Grayscale Digital Images of Fabric Specimens
Cotton	M1 *	** 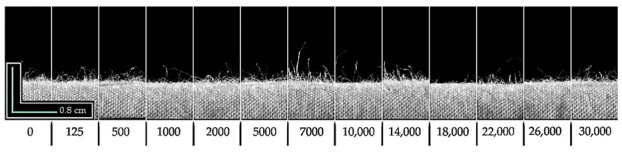 **
	M2 **	** 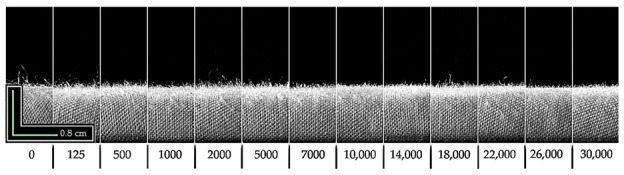 **
Wool	M1 *	** 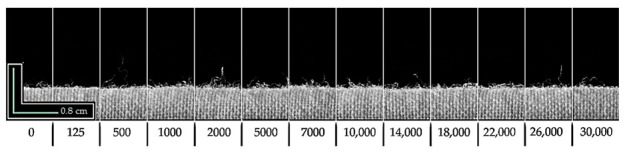 **
	M2 **	** 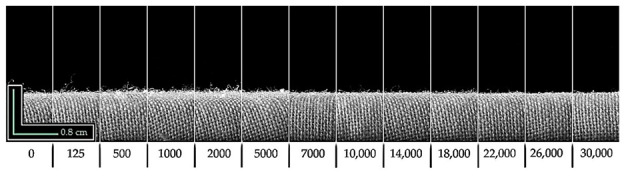 **
Viscose	M1 *	** 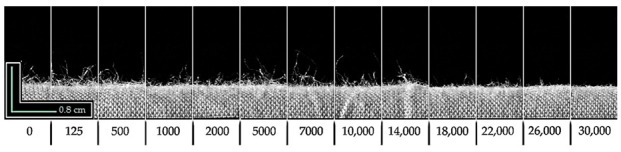 **
	M2 **	** 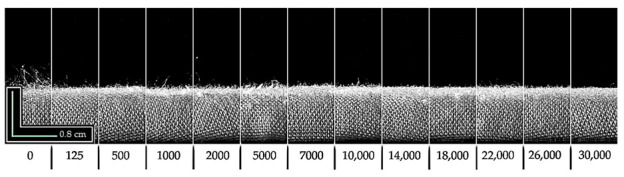 **
Polyamide 6.6	M1 *	** 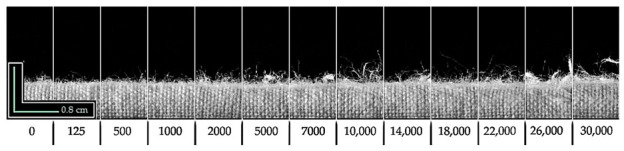 **
	M2 **	** 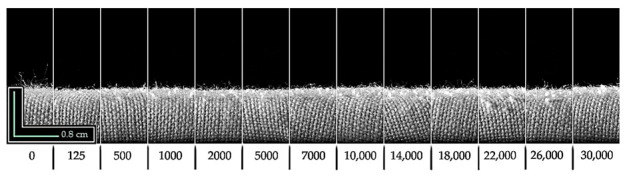 **
Polyester	M1 *	** 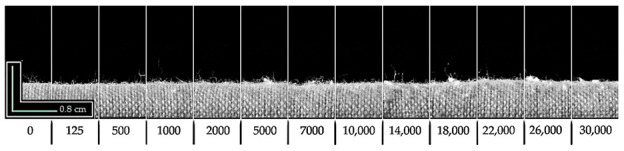 **
	M2 **	** 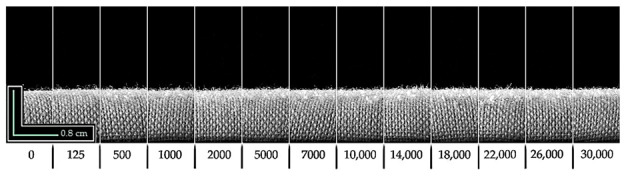 **
Acrylic	M1 *	** 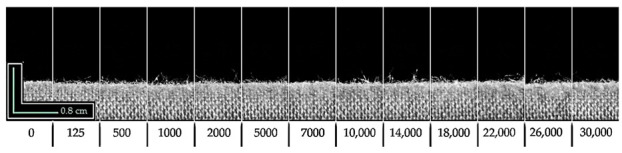 **
	M2 **	** 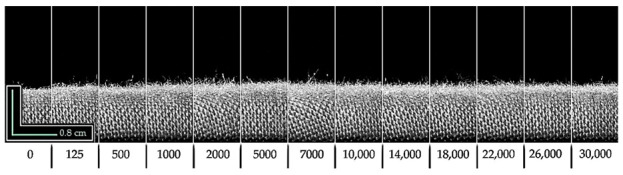 **

* The ICI rotating box method; ** Martindale method.

**Table 6:** Arithmetic mean ($\bar{t_{fl}}$) and corresponding coefficient of variation (*V*) of the surface hairiness layer for non-abraded fabric specimens (0 control point) and fuzzing layer height for abraded fabric specimens (125 to 30,000 control points), determined by the instrumental assessment method.

Control Points	Arithmetic Mean of Hairiness and Fuzzing Layer Height, mm/Coefficient of Variation, %					
	Cotton		Wool		Viscose	
	M1 *	M2 **	M1 *	M2 **	M1 *	M2 **
0	2.07/56.38	2.50/51.59	1.46/41.72	1.21/54.18	1.71/50.70	2.09/54.62
125	1.98/37.70	2.17/36.55	0.70/61.90	1.54/40.33	2.45/54.29	1.74/34.76
500	1.10/60.81	1.77/36.44	0.92/67.37	1.67/36.12	1.21/63.88	1.40/38.80
1000	1.48/49.22	1.79/30.04	0.90/83.56	1.46/41.69	1.18/84.48	1.78/34.39
2000	1.66/49.97	2.07/39.13	0.80/72.08	1.38/45.12	1.31/94.04	1.71/48.33
5000	1.78/57.72	1.94/38.21	0.43/80.88	1.17/38.82	2.39/58.95	1.93/30.64
7000	1.93/57.38	1.71/35.41	1.14/72.71	1.03/38.65	1.41/67.69	1.40/33.86
10,000	1.79/46.48	1.74/36.19	1.10/42.82	1.10/35.44	0.8/107.75	1.51/37.04
14,000	1.59/68.10	1.71/30.77	0.72/69.63	1.03/40.77	0.75/82.29	1.38/35.56
18,000	0.51/87.86	1.97/30.94	0.82/58.60	0.92/51.20	0.77/69.84	1.32/35.69
22,000	0.97/81.55	1.59/35.94	0.55/78.37	1.02/39.55	0.67/69.30	1.74/36.67
26,000	1.79/43.54	1.36/39.98	0.79/69.06	0.95/30.43	0.52/62.35	1.32/33.05
30,000	1.36/58.38	1.56/35.14	0.92/52.88	0.82/49.24	0.32/84.31	1.11/34.74
**Control Points**	**Arithmetic Mean of Hairiness and Fuzzing Layer Height, mm/Coefficient of Variation, %**					
	**Polyamide 6.6**		**Polyester**		**Acrylic**	
	M1 *	M2 **	M1 *	M2 **	M1 *	M2 **
0	1.51/43.90	1.78/38.58	0.68/48.40	0.84/42.31	0.70/45.60	0.89/42.63
125	1.04/83.34	1.47/39.96	0.80/70.22	1.49/41.46	0.94/71.90	1.55/42.14
500	0.80/79.63	1.74/27.83	0.53/93.98	1.34/33.37	1.15/43.85	1.63/43.63
1000	1.14/51.80	1.82/38.78	0.79/65.10	1.25/36.12	0.65/74.45	1.96/34.76
2000	1.87/35.73	1.58/31.09	1.04/46.15	1.67/30.55	1.31/46.65	2.07/38.10
5000	1.72/43.19	1.46/36.67	0.71/52.26	1.51/25.54	0.76/76.95	1.46/29.43
7000	1.46/50.60	1.75/28.55	0.86/52.58	1.33/31.79	0.77/59.99	2.38/27.04
10,000	2.37/41.06	1.92/27.62	0.74/55.82	1.34/33.18	1.02/47.37	1.92/26.58
14,000	1.49/48.19	1.76/33.61	1.18/50.11	1.77/32.87	0.97/43.21	1.77/31.17
18,000	2.63/36.56	1.68/29.03	0.93/54.78	1.36/33.14	1.26/49.24	1.67/37.89
22,000	1.99/41.13	1.48/32.15	1.31/46.90	1.37/34.79	1.07/43.92	1.72/33.79
26,000	3.73/35.49	1.55/36.12	1.28/45.32	1.28/29.47	0.65/68.84	1.52/34.09
30,000	2.98/33.80	1.89/29.53	0.94/48.58	1.39/31.35	0.68/63.11	1.64/28.83

* The ICI rotating box method; ** Martindale method.

**Table 7:** Median ($\tilde{t_{fl}}$) surface fuzzing layer height for non-abraded fabric specimens (at 0 control point, corresponding to surface hairiness) and abraded fabric specimens (after 125 to 30,000 control points, corresponding to surface fuzzing), as determined by the instrumental assessment method.

Control Points	Median of Fuzzing Layer Height, mm											
	Cotton		Wool		Viscose		Polyamide 6.6		Polyester		Acrylic	
	M1 *	M2 **	M1 *	M2 **	M1 *	M2 **	M1 *	M2 **	M1 *	M2 **	M1 *	M2 **
0	1.79	2.35	1.32	1.13	1.52	2.00	1.45	1.78	0.50	0.86	0.64	0.82
125	1.87	2.13	0.58	1.58	2.11	1.78	0.78	1.60	0.63	1.55	0.89	1.56
500	0.98	1.91	0.80	1.65	1.15	1.48	0.63	1.78	0.41	1.41	1.08	1.64
1000	1.35	1.91	0.73	1.50	1.00	1.78	1.00	1.93	0.70	1.26	0.56	2.01
2000	1.50	2.20	0.65	1.43	1.00	1.70	1.82	1.63	0.93	1.70	1.23	2.16
5000	1.65	1.91	0.36	1.21	1.96	2.00	1.67	1.48	0.70	1.55	0.64	1.49
7000	1.72	1.76	0.95	1.06	1.22	1.48	1.23	1.78	0.78	1.41	0.71	2.45
10,000	1.65	1.83	1.02	1.06	0.55	1.63	2.19	2.00	0.70	1.41	0.93	1.93
14,000	1.35	1.83	0.58	0.99	0.63	1.48	1.37	1.78	1.00	1.85	0.93	1.79
18,000	0.39	2.05	0.73	0.91	0.63	1.41	2.48	1.63	0.85	1.41	1.23	1.64
22,000	0.76	1.68	0.43	0.99	0.55	1.78	1.89	1.56	1.15	1.41	1.01	1.79
26,000	1.65	1.46	0.73	0.99	0.48	1.41	3.74	1.63	1.22	1.33	0.56	1.56
30,000	1.20	1.68	0.88	0.84	0.26	1.11	2.86	1.93	0.85	1.48	0.64	1.64

* The ICI rotating box method; ** Martindale method.

**Table 8:** The percentage change (∆$\tilde{t_{fl}}$) in the median surface fuzzing layer height calculated according to Equation (2) for abraded fabric specimens (after 125 to 30,000 control points, corresponding to surface fuzzing), as determined by the instrumental assessment method.

Control Points	The Percentage Change of the Median Fuzzing Layer Height, %											
	Cotton		Wool		Viscose		Polyamide 6.6		Polyester		Acrylic	
	M1 *	M2 **	M1 *	M2 **	M1 *	M2 **	M1 *	M2 **	M1 *	M2 **	M1 *	M2 **
125	4.5	−9.4	−56.1	39.8	38.8	−11.0	−46.2	−10.1	26.0	80.2	39.1	90.2
500	−45.3	−18.7	−39.4	46.0	−24.3	−26.0	−56.6	0.0	−18.0	64.0	68.8	100.0
1000	−24.6	−18.7	−44.7	32.7	−34.2	−11.0	−31.0	8.4	40.0	46.5	−12.5	145.1
2000	−16.2	−6.4	−50.8	26.5	−34.2	−15.0	25.5	−8.4	86.0	97.7	92.2	163.4
5000	−7.8	−18.7	−72.7	7.1	28.9	0.0	15.2	−16.9	40.0	80.2	0.0	81.7
7000	−3.9	−25.1	−28.0	−6.2	−19.7	−26.0	−15.2	0.0	56.0	64.0	10.9	198.8
10,000	−7.8	−22.1	−22.7	−6.2	−63.8	−18.5	51.0	12.4	40.0	64.0	45.3	135.4
14,000	−24.6	−22.1	−56.1	−12.4	−58.6	−26.0	−5.5	0.0	100.0	115.1	45.3	118.3
18,000	−78.2	−12.8	−44.7	−19.5	−58.6	−29.5	71.0	−8.4	70.0	64.0	92.2	100.0
22,000	−57.5	−28.5	−67.4	−12.4	−63.8	−11.0	30.3	−12.4	130.0	64.0	57.8	118.3
26,000	−7.8	−37.9	−44.7	−12.4	−68.4	−29.5	157.9	−8.4	144.0	54.7	−12.5	90.2
30,000	−33.0	−28.5	−33.3	−25.7	−82.9	−44.5	97.2	8.4	70.0	72.1	0.0	100.0

* The ICI rotating box method; ** Martindale method.

**Table 9:** Fuzz grades assigned according to nine grading classes defined by the percentage change in the median fuzzing layer height (presented in Table 3) for abraded fabric specimens (after 125 to 30,000 control points, corresponding to surface fuzzing), as determined by the instrumental assessment method (*FG_DA_*).

Control Points	Fuzz Grades—Advanced Digital Assessment Method											
	Cotton		Wool		Viscose		Polyamide 6.6		Polyester		Acrylic	
	M1 *	M2 **	M1 *	M2 **	M1 *	M2 **	M1 *	M2 **	M1 *	M2 **	M1 *	M2 **
125	5.0	5.0	5.0	3.5	3.5	5.0	5.0	5.0	4.0	1.5	3.5	1.0
500	5.0	5.0	5.0	3.0	5.0	5.0	5.0	5.0	5.0	2.5	2.0	1.0
1000	5.0	5.0	5.0	4.0	5.0	5.0	5.0	5.0	3.5	3.0	5.0	1.0
2000	5.0	5.0	5.0	4.0	5.0	5.0	4.0	5.0	1.5	1.0	1.0	1.0
5000	5.0	5.0	5.0	5.0	4.0	5.0	4.5	5.0	3.5	1.5	5.0	1.5
7000	5.0	5.0	5.0	5.0	5.0	5.0	5.0	5.0	2.5	2.5	5.0	1.0
10,000	5.0	5.0	5.0	5.0	5.0	5.0	3.0	4.5	3.5	2.5	3.0	1.0
14,000	5.0	5.0	5.0	5.0	5.0	5.0	5.0	5.0	1.0	1.0	3.0	1.0
18,000	5.0	5.0	5.0	5.0	5.0	5.0	2.0	5.0	2.0	2.5	1.0	1.0
22,000	5.0	5.0	5.0	5.0	5.0	5.0	4.0	5.0	1.0	2.5	2.5	1.0
26,000	5.0	5.0	5.0	5.0	5.0	5.0	1.0	5.0	1.0	3.0	5.0	1.0
30,000	5.0	5.0	5.0	5.0	5.0	5.0	1.0	5.0	2.0	2.0	5.0	1.0

* The ICI rotating box method; ** Martindale method.

## Data Availability

The original contributions presented in this study are included in the article. Further inquiries can be directed to the corresponding author.

## Abbreviations

The following abbreviations are used in this manuscript:

*D*	Distance between the highest and lowest white pixel along the vertical axis (y-direction)
*t_fl_*	Hairiness or fuzzing layer; height of the hairiness (non-abraded) or fuzzing (abraded) layer on the fabric specimen’s surface
*h*_1_	Distance from the visible side edge of the specimen holder to the top of the holder with felt underlay for the rotating box specimen holder
*h*_2_	Distance from the visible side edge of the specimen holder to the top of the holder with felt underlay for the Martindale method specimen holder
*t_m_*	Fabric layer = fabric thickness
∆$\tilde{t_{fln}}$	Percentage change in the median fuzzing layer height
$\tilde{t_{fln}}$	Median fuzzing layer height of the abraded fabric specimen
$\tilde{t_{fl0}}$	Median hairiness layer height of the non-abraded fabric specimen
*n*	Number of control points (number of ICI box revolutions or Martindale fuzzing rubs)
M1	The ICI rotating box method
M2	Martindale method
*FG_VA_*	Fuzz grades—standard visual assessment method
*FG_DA_*	Fuzz grades—advanced digital assessment method
*p*	Probability value
